# Prevalence and patient related factors associated with Extended-Spectrum Beta-Lactamase producing *Escherichia coli* and *Klebsiella pneumoniae* carriage and infection among pediatric patients in Tanzania

**DOI:** 10.1038/s41598-021-02186-2

**Published:** 2021-11-23

**Authors:** Nuru Letara, James Samwel Ngocho, Nahid Karami, Sia E. Msuya, Balthazar Nyombi, Nancy A. Kassam, Susann Skovbjerg, Christina Åhren, Rune Philemon, Blandina T. Mmbaga

**Affiliations:** 1 Department of Pediatric and Child Health, Kilimanjaro Christian Medical Center, Moshi, Tanzania; 2 Kilimanjaro Christian Medical University College, Moshi, Tanzania; 3 Department of Clinical Microbiology, Sahlgrenska University Hospital, Region Västra Götaland, Gothenburg, Sweden; 4 Department of Infectious Diseases, Institute of Biomedicine, Sahlgrenska Academy, University of Gothenburg, Gothenburg, Sweden; 5 Centre for Antibiotic Resistance Research (CARe), University of Gothenburg, Gothenburg, Sweden; 6 Kilimanjaro Clinical Research Institute, Moshi, Tanzania

**Keywords:** Bacterial infection, Gastrointestinal diseases, Infectious diseases

## Abstract

Extended-Spectrum Beta-Lactamase (ESBL) producing *Enterobacteriaceae* (EPE) is increasing worldwide, though less documented in low-income settings. Here we determined the prevalence of EPE infection and carriage, and patient factors associated with EPE-carriage among pediatric patients in three health care levels in Tanzania. Between January and April 2016, 350 febrile children (median age 21 months) seeking care at a university or a regional referral hospital, or a health centre in Moshi municipality, Tanzania, were included. Socio-demographic characteristics were collected using a questionnaire. Rectal swabs and blood cultures were collected from all children (*n* = 350) and urinary samples from 259 children at admission. ESBL-phenotype and antimicrobial susceptibility were determined for *Klebsiella pneumoniae (K. pneumoniae)* and *Escherichia coli (E. coli*) isolates. Only one EPE case (*E. coli*) in blood and four in urine (one *E. coli* and three *K. pneumoniae*) were found, whereas (*n* = 90, 26%) of the children were colonized in feces (ESBL-*E. coli*; *n* = 76, ESBL-*K. pneumoniae, n* = 14). High resistance rates were seen in fecal ESBL-*E. coli* (*n* = 76) against trimethoprim-sulfamethoxazole (*n* = 69, 91%), gentamicin (*n* = 51, 67%), ciprofloxacin (*n* = 39, 51%) and chloramphenicol (*n* = 27, 35%) whereas most isolates were sensitive to amikacin (*n* = 71, 93%). Similar rates were seen for fecal ESBL-*K. pneumoniae*. Resistance to first line antibiotics were also very high in fecal *E. coli* not producing ESBL. No sociodemographic factor was associated with EPE-carriage. Children colonized with EPE were younger than 12 months (*n* = 43, 48%) and often treated with antibiotics (*n* = 40, 44%) in the previous two months. After adjustment for age children admitted to the intensive care unit had higher odds of EPE fecal carriage compared with those in the general wards (OR = 3.9, 95%CI = 1.4–10.4). Despite comparatively high rates of fecal EPE-carriage and previous antibiotic treatment, clinical EPE cases were rare in the febrile children. The very high resistant rates for the EPE and the non-ESBL producing *E. coli* to commonly used antibiotics are worrying and demand implementation of antibiotic stewardship programs in all levels of health care in Tanzania.

## Introduction

The prevalence of Extended-Spectrum Beta-Lactamase (ESBL) producing *Enterobacteriaceae* (EPE) is increasing worldwide and varies between different geographical areas. In Eastern Africa the prevalence of EPE varies from 6 to 17% in the community and 38–83 % in hospital settings^1^. In a Tanzanian study, performed in Dar es salaam in 2011 the prevalence of fecal carriage of EPE was found to be 50% among children admitted to a tertiary hospital and 12% among healthy community children^2^. Intestinal carriage of EPE was found in 32% of street children, who live in urban Mwanza^3^. Neonatal EPE sepsis was detected in 10% of the neonates investigated in Tanzania^4^. A wide range of factors contributes to carriage rates of EPE in the community^5,6^. The use of antibiotics in the last three months was associated with faecal carriage of EPE among food handlers in the West Coast Region of The Gambia^7^, and an association between antimicrobial use and ESBL colonization has also been shown from a range of countries and settings in Sub-Saharan Africa^8^.

Within the hospital settings differences in patient care and infection control measures are factors that contribute to differences in the prevalence of EPE^9,10^. Patients with EPE- infections have higher mortality rates and require longer hospital stay than those not infected with ESBL-producing bacteria^11–15^. ESBL-producing bacteria confer resistance to penicillins and most cephalosporins and are often co-resistant to other antimicrobial classes such as, trimethoprim-sulfamethoxazole and quinolones and increasingly also to aminoglycosides, thus limiting treatment options^17–19^.

The prevalence of EPE is largely unknown in the Moshi municipality, Tanzania. At the time of the study there were no diagnostic or screening tools in place in the routine care and limited options of antibiotics available for treatment. The current study was designed to determine the prevalence of EPE clinical infections and carriage resistance patterns in fecal isolates as well as factors associated with carriage of ESBL producing *E. coli* and *K. pneumoniae* among pediatric patients at different health care levels in the Moshi municipality.

## Material and methods

### Design, setting and sampling

In this facility-based cross-sectional study performed between January and April 2016, 350 children between 2 months and 15 years with fever (≥ 37.5 °C) seeking care at Kilimanjaro Christian Medical Centre (KCMC) (*n* = 150), Mawenzi regional referral hospital (*n* = 100) or Pasua health centre (*n* = 100) in the Moshi municipality, Kilimanjaro region, Northern Tanzania, were included. KCMC is a zonal referral hospital, receiving patients from the five regions of northern Tanzania and hence provides care for approximately 15 Million people. Mawenzi Regional Referral Hospital is a referral hospital for the Kilimanjaro region, receiving referrals from the seven districts of Kilimanjaro region hence about 2 million inhabitants, while Pasua health center is the primary health care facility serving people living in Pasua ward. Rectal swabs and blood cultures were obtained from all children at admission and urine cultures from 259 of the 350 participants, while 91 participants had no urine culture.

The cohort whose urine were not cultured (*n* = 91) were hospitalized with the following clinical diagnosis; meningitis (*n* = 21), pneumonia (*n* = 20), septicemia (*n* = 16), urinary tract infection (*n* = 12), bronchitis (*n* = 4), acute tonsillitis (*n* = 3), acute otitis media (*n* = 3), chronic malnutrition (*n* = 5) and malaria (*n* = 7). They were cared for at KCMC (*n* = 53), Mawenzi RRH (*n* = 22) and Pasua HC (*n* = 16).

Face to face interviews were conducted using a questionnaire with parents/guardians. In addition to the demographics (age, sex and place of residence, level of education), information was collected on household size, source of water, and recent contact with the health system as well as previous history of medical care.

### Bacterial cultures

Cultures were obtained at admission. A rectal swab was taken and placed in sterile Amies transport medium (Eswab, Copan Diagnostics Inc., Murrieta, USA). Urine was collected in a sterile container and 1–3 mL of blood were drawn for blood culture using a BD Bactec Plus Aerobic/F blood culture bottle (Becton Dickinson and Company, MD, USA). The specimens were transported to the Department of Microbiology laboratory at KCMC hospital and processed immediately. Samples from urine and blood culture bottles were cultured according to routine procedure including culture on MacConkey agar (Oxoid Limited, Basingstoke, Hampshire, UK) to detect Gram negative rods. Plates were incubated aerobically at 37 °C for 16–18 h. Fecal cultures were streaked on MacConkey agar plates. A cefuroxime disc (CXM 30 µg, Oxoid Ltd) was applied on the solid growth of the culture to detect EPE isolates corresponding to that of European Committee on Antimicrobial Susceptibility Testing (EUCAST) (http://www.eucast.org/clinical_breakpoints/). The plates were incubated in 37 °C aerobically overnight. A cefuroxime zone < 22 mm indicated possible ESBL-production. Up to three *E. coli*- or Klebsiella-like colonies were picked from within the cefuroxime zones or nearest to the zone edge and re-cultivated on a new MacConkey agar plate with a CXM disc for subsequent analyses of ESBL-phenotype. *E. coli* and *K. pneumoniae* species determination was based on morphology, lactose fermentation, indole production and motility test. Isolates not identified as *E. coli* or *K. pneumoniae* were not included in the subsequent EPE-analyses. In addition, *E. coli* isolates (one per child) were collected from 260 fecal samples with no bacteria growing adjacent to the cefuroxime disc.

### Antibiotic susceptibility testing

**Figure 1:**
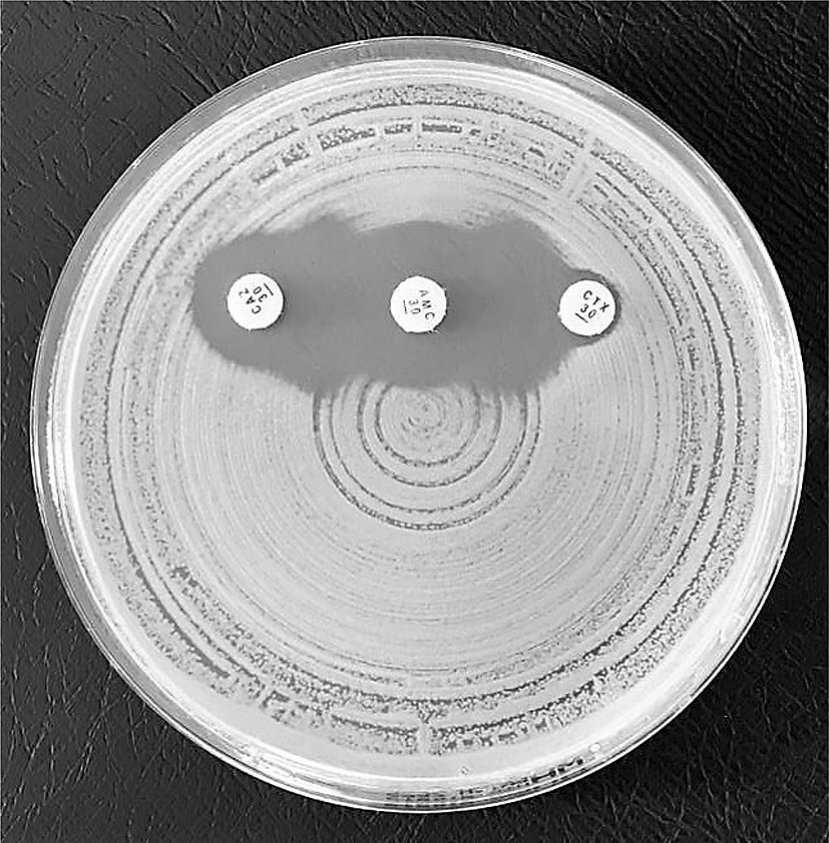
Image showing double-disk diffusion method (photo by Nahid Karim).

Cefuroxime resistant *E. coli* and *K. pneumoniae* isolates were screened for ESBL-production by the double disc synergy test using ceftazidime (30 µg), cefotaxime (30 µg) and amoxicillin/clavulanate (30 µg discs) (Oxoid Ltd) as the inhibitory substance. Test was considered positive for ESBL when there was a synergy between any two antibiotics (Fig. 1) with amoxicillin/clavulanate.

Susceptibility for eleven antibiotics (ampicillin, chloramphenicol, amikacin, ciprofloxacin, trimethoprim-sulfamethoxazole, gentamicin, piperacillin-tazobactam, amoxicillin-clavulanate, ceftazidime, ceftriaxone, cefuroxime and meropenem) in all ESBL-positive fecal isolates and a subset of arbitrary selected non-ESBL producing fecal *E coli* was determined using the disc diffusion method (Oxoid Ltd) and breakpoints according to the European Committee on Antimicrobial Susceptibility Testing (EUCAST) guidelines at the time of the study. Resistant and intermediate isolates were considered as resistance.

### Statistics

Data from the questionnaires were entered and analyzed using statistical package of social sciences version 21.0. Descriptive statistics was used to summarize the data, frequency and percentage for categorical variables and measures of central tendency and their dispersion for numerical data. A logistic regression analysis was carried out to examine the factors associated with EPE carriage. In the bivariate analysis, factors with a *p*-value ≤ 0.05 were included in the final multivariable model. For categorical variables, the Chi-squared test was used. Differences with a p-value less than 0.05 were considered statistically significant.

### Ethical consideration

All methods were carried out in accordance with the study protocol and the good clinical practice standards. Ethical permission was received from Kilimanjaro Christian Medical University College, with Ethical clearance No.865. Parents/caretaker signed informed consent, confidentiality was maintained, and the study used unique ID codes for study participants.

## Results

### Sociodemographic and clinical data for all children

Parents or guardians to a total of 350 eligible children were approached and they all accepted their child to be enrolled in this study. The median age of the participants was 21 months (IQR 8–47 months), a majority (69%) were less than 3 years old, 55% (*n* = 194) were boys and 67% (*n* = 236) were living in the Moshi urban area. Additional sociodemographic characteristics are shown in Table 1.

**Table 1:** General characteristics for those colonized with Extended-spectrum beta-lactamase (ESBL) producing *Enterobacteriaceae* (EPE) in feces at admission.

**Variables**	No. of children (%)	
	All included (*N* = 350)	EPE colonized (*N* = 90)
**Sex**		
Male	194 (55)	52 (58)
Female	156 (45)	38 (42)
**Age in months**		
Median (IQR)	21 (8–47)	12 (6–31)
**Age categories**		
2–11	121 (35)	43 (48)
12–23	82 (23)	17 (19)
24–35	40 (11)	6 (7)
36–59	38 (11)	10 (11)
60 and above	69 (20)	14 (15)
**Residence**		
Moshi urban	236 (67)	59 (65)
Moshi rural	52 (15)	15 (17)
Other	62 (18)	16 (18)
**Household size**		
2–3	65 (19)	17 (19)
4–5	170 (49)	45 (50)
More than 5	115 (33)	28 (31)
**Source of drinking water**		
Tap	327 (93)	83 (92)
Well	19 (5)	5 (6)
Other	4 (1)	2 (2)
Healthcare facility attended*		
KCMC	150 (43)	53 (59)
Mawenzi RRH	100 (29)	23 (26)
Pasua HC	100 (29)	14 (15)
**Education level**		
Not in school	254 (73)	68 (76)
Pre-primary	48 (14)	14 (15)
Primary	48 (14)	8 (9)
**Ward admitted**		
General	332 (95)	80 (89)
Pediatric ICU**	15 (4)	9 (10)
Surgical ICU	3 (1)	1(1)
**Length of hospital stay after admission**		
≤ 7 days	257 (73)	65 (72)
8 – 14 days	40 (11)	11 (120
> 14 days	53 (15)	14 (16)
**History of previous surgery**		
Yes	10 (3)	5 (6)
No	340 (97)	85 (94)
**Antibiotic use previous 2 months**		
Yes	125 (36)	40 (44)
No	225 (64)	50 (56)
**History of previous admission**		
Yes	106 (30)	33 (37)
No	244 (70)	57 (63)
**HIV/AIDS status**		
Positive	7 (2)	2 (2)
Negative	343 (98)	88 (98)

*KCMC Kilimanjaro Christian Medical Centre, *Mawenzi RRH* Mavenzi Regional Referral Hospital, *Pasua HC* Pasua Health Cent.

**ICU Intensive Care Unit.

Most participants (*n* = 332, 95%) were admitted to a general pediatric ward. Intensive care units (ICU) were only available at KCMC. Most children (*n* = 257, 73%) had a subsequent hospital stay of less or equal to 7 days and the majority (*n* = 244, 70%) had no history of previous admission to any health care facility as shown in Table 1. Most children (*n* = 317, 91%) had mild or moderate fever (≥ 37.5 °C).

### Clinical culture results

Out of the 350 children in whom blood cultures were performed, only one child (11 months) had EPE (*E. coli*) in blood and was EPE-positive in stool as well. This child was admitted to the ICU at KCMC due to burn wounds and malnutrition. In addition, positive blood cultures were detected in 34 children, the dominating pathogens were *S. aureus* (*n* = 15, 44%), *E. coli* (*n* = 7, 21%) and *K. pneumoniae* (*n* = 4, 12%).

A positive urine culture was found in 9% (23 out of the 259) of sampled children; 13 (5%) with *E. coli* and 10 (4%) with *K. pneumoniae*. Of these, four (17%) had EPE in urine; one child with *E. coli* (from Pasua HC), and three with *K. pneumoniae* (one from KCMC and two from Pasua HC). Two of these four children carried EPE (*E. coli*) in feces at admission as well. No child was positive for EPE in both urine and blood. Resistance rates for the clinical EPE-isolates were not significantly different from those in stool EPE.

Results from the rectal swabs showed that 26% of (90/350) children carried EPE, i.e. 76(22%) ESBL-*E. coli* (KCMC; 46 (13%), Mawenzi RRH;18 (5%), Pasua HC; 12 (3%) and 14 (4%) ESBL- *K. pneumoniae* (KCMC;7 (2%), Mawenzi RRH; 5 (1%), Pasua HC; 2 (1%) in feces. None carried EPE of both species. In addition, 17 (5%) children carried cefuroxime-resistant Gram-negative bacteria of other species than *E. coli* or *K. pneumoniae*. These isolates were not further typed according to predefined inclusion criteria of EPE limited to *E coli* and *K. pneumoniae*.

The resistance rates for colonized ESBL-*E. coli* and ESBL-*K. pneumoniae*, respectively, were high against trimethoprim-sulfamethoxazole (*n* = 69, 91% and *n* = 14, 100%), gentamicin (*n* = 51, 67% and *n* = 10, 71%), ciprofloxacin (*n* = 39, 51% and *n* = 9, 64%) and chloramphenicol (*n* = 27, 35% and *n* = 4, 29%). Only one (1%) of the EPE-isolates, being *E. coli* was resistant to meropenem. In addition to ESBL-phenotype, co-resistance was prevalent in both ESBL-*E. coli* and ESBL-*K. pneumoniae*, i.e., 50% (*n* = 38) and 64% (*n* = 9) to trimethoprim-sulfamethoxazole + ciprofloxacin and 37% (*n* = 28) and 43% (*n* = 6) to trimethoprim-sulfamethoxazole + ciprofloxacin- + gentamycin, respectively. In 40 (53%) ESBL-*E. coli* and 9 (64%) ESBL-*K. pneumoniae* isolates, amikacin, piperacillin-tazobactam, meropenem or chloroamphenicol were the only sensitive alternatives.

Resistance rates in ESBL-*E. coli* were similar in all settings even though resistance to gentamicin and co-resistance were more common in isolates from children admitted to the tertiary referral hospital (Table 2).

**Table 2:** Non-susceptibility against various antibiotics in ESBL-producing *E. coli* from 76 children detected in feces at admission in relation to level of care, i.e., zonal referral hospital (KCMC), regional referral hospital (Mawenzi RRH) and local health center (Pasua HC).

Antibiotic	KCMC *(N* = 46)	Mawenzi RHH (*N* = 18)	Pasua HC (*n* = 12)
	*N* (%)*	*N* (%) *	*N* (%) *
Ampicillin	46 (100)	18 (100)	12 (100)
Amoxicillin-clavulanic acid	46 (100)	16 (89)	11 (92)
Trimethoprim-sulfamethoxazole((TS)	45 (98)	15 (83)	9 (75)
Ceftazidime	46 (100)	18 (100)	12 (100)
Ceftriaxone	46 (100)	18 (100)	12 (100)
Meropenem	1 (2)	0 (0)	0 (0)
Piperacillin-tazobactam	9 (19)	1 (1)	4 (33)
Ciprofloxacin	27 (59)	8 (44)	4 (33)
Gentamicin	35 (76)	11 (61)	5 (42)
Amikacin	5 (11)	0 (0)	0 (0)
Chloramphenicol	16 (35)	7 (39)	4 (33)
TS + CI	27 (59)	20 (44)	3 (25)
TS + CI + GM	21 (46)	5 (28)	2 (17)

*Percentage of non-susceptibility.

*TS* trimethoprim-sulfamethoxazole, *CI* ciprofloxacin, *GM* gentamicin.

We also compared resistance rates in 278 fecal *E. coli* isolates with and without ESBL-production (Table 3). Resistance rates were lower in the non-ESBL-producing isolates. However, resistance rates to commonly used antibiotics including amoxicillin, amoxicillin-clavulanic acid and trimethoprim-sulfamethoxazole were very high and within the same range as for the ESBL-producing isolates. Five isolates, including one EPE-isolate and one multidrug resistant isolate (resistant to trimethoprim-sulfamethoxazole, gentamycin and amikacin) had reduced susceptibility to meropenem but none of these isolates were further typed for carbapenemase genes. All five children had been hospitalized the previous months.

**Table 3:** Non-susceptibility against various antibiotics in ESBL-producing *E. coli* and in *E. coli* not producing ESBL, detected in feces in 278 *E. coli* isolates from 350 children at admission.

Antibiotic	Number of cases (%)*	
	ESBL-*E. coli* (*n* = 76)	Non-ESBL-*E. coli* (*n* = 202)
Ampicillin	76 (100)	202 (100)
Amoxicillin-clavulanic acid	73 (96)	166 (82)
Trimethoprim-sulfamethoxazole	69 (91)	150 (74)
Gentamicin	51 (67)	27 (13)
Ceftazidime	76 (100)	16 (8)
Ceftriaxone	76 (100)	17 (8)
Meropenem	1 (1)	4 (2)
Piperacillin-tazobactam	14 (18)	18 (9)
Amikacin	5 (7)	7 (3)
Chloramphenicol	27 (35)	35 (17)
Ciprofloxacin	39 (51)	15 (7)
TS + CI	38 (50)	12 (6)
TS + CI + GM	28 (37)	5 (2)

*Percentage of non-susceptibility.

*TS* trimethoprim-sulfamethoxazole, *CI* ciprofloxacin, *GM* gentamicin.

Thirty six percent (*n* = 125) of the children had used antibiotics 2 months before medical care of which ampicillin/amoxicillin alone or in combination with another drug, mostly gentamycin, was the most common drug noted with no differences in those EPE colonized or not.

### Sociodemographic factors and clinical admission data in relation to EPE-carriage

There were generally very few differences in sociodemographic factors or previous medical history between those colonized and not colonized with EPE in feces at admission (Table 1). Higher rates of EPE-carriage were seen in those admitted to the zonal referral hospital (53/144, 37%) as compared to regional referral hospital (23/94, 23%) and the rural health center (14/95, 15%) (Table 1). Children less than one year of age were significantly more often colonized than older children (36% versus 21%, *p* = 0.003). However, previous admission rates were higher at KCMC (81/150, 54%) as compared to Mawenzi RRH (18/100, 18%) and Pasua HC (7/100, 7%) as were previous antibiotic treatment (KCMC: 77/150, 51%, Mawenzi RRH; 22/100, 22%, Pasua HC; 26/100, 26%). In those less than one year of age and admitted to KCMC, 59% (39/66) had been given antibiotics prior to admission and 51% (34/66) had been admitted previously.

In the unadjusted analysis, prior antibiotic use was associated with ESBL carriage (Table 4). There was no evident difference in given antibiotics to those colonized with EPE compared to non-EPE carriers. Although a variety of antibiotics had been administered, ampicillin/amoxicillin alone or in combination with other drugs were predominant in both groups. In unadjusted analysis, intensive care unit admittance was also associated with ESBL carriage (Table 4). Those admitted to ICU had 3.7 higher odds of ESBL carriage as compared with those in the general ward (OR = 3.7, 95%CI = 1.4–9.6). After adjusting for age, the only factor remaining significant was ward, i.e., those admitted to ICU had 3.9 higher odds of ESBL carriage compared with those in the general ward (OR = 3.9, 95%CI = 1.4–10.4) (Table 5).

**Table 4:** Bivariable analysis of factor associated with carriage of Extended-spectrum beta-lactamase (ESBL) producing Enterobacteriaceae (EPE) in feces at admission.

Variable	Total (*n* = 333)	ESBL (*n* = 90)	Crude OR (95%CI	*p*-value
**Ward admitted**				
General	315	80 (25)	Ref	
ICU	18	10 (56)	3.7 (1.4 – 9.6)	0.008
**Length of hospital stay after admission (days)**				
1- 7 days	245	65 (27)	Ref	
8 or more days	88	25 (28)	1.1 (0.6–1.9)	0.734
**History of surgery**				
No	323	85 (26)	ref	
Yes	10	5 (50)	2.8 (0.8–9.9)	0.110
**Previous antibiotic**				
No	216	50 (23)	Ref	
Yes	117	40 (34)	1.7 (1.1–2.8)	0.031
**Previous admission**				
No	234	57 (24)	Ref	
Yes	99	33 (33)	1.5 (0.9–2.6)	0.093
**HIV/AIDS status**				
No	327	88 (27)	Ref	
Yes	6	2 (33)	1.4 (0.2–7.5)	0.727
**Healthcare facility attended**				
Pasua HC	95	14 (15)	Ref	
Mawenzi RRH	94	23 (25)	1.9 (0.9–3.9)	0.095
KCMC	144	53 (37)	3.4 (1.7–6.5)	< 0.001

**Table 5:** Multivariable analysis of factor associated with carriage of Extended-spectrum beta-lactamase (ESBL) producing Enterobacteriaceae (EPE) in feces at admission.

Variable	Adjusted OR (95%CI	*p*-value
**Ward admitted**		
General	Ref	
ICU	3.9 (1.4–10.4)	0.007
**Previous antibiotic**		
No	Ref	
Yes	1.6 (0.9–2.6)	0.090

Odds ratio adjusted for age in months and sex.

## Discussion

This cross-sectional study was designed to determine the prevalence of fecal carriage and clinical infection with ESBL-producing *E. coli* and *K. pneumoniae* among children admitted with fever to three different health care levels in Moshi Municipality in Tanzania. Fecal carriage rates were related to sociodemographic as well as to medical history and preadmission data gathered by face-to-face interviews following questionnaire administration. Clinical infection in blood or urine was detected by culture in 57 children but only five of them were due to EPE. Infections related to EPE were rare despite a rather high EPE fecal carriage rate, especially in those admitted to the zonal referral hospital including the ICU wards. This is in line with more recent findings that patients gut colonized with EPE rarely develop subsequent EPE-infections, unless part of selected subgroups, like patients in neonatal ICU (NICU), ICU or with hematological malignancies^4,20,21^.

Also, correlation between gut colonization and clinical infection is more often seen with ESBL*-K. pneumoniae* than ESBL- *E. coli*. This indicates that the correlation primarily applies to certain subsets of patients, including those with other comorbidities or immunodeficiencies^4^.

No sociodemographic factor was found to correlate with EPE-carriage. Comparatively high rates (32%) were reported from street children in a Tanzanian city^3^, and poverty, sharing beds, overcrowded households have previously been reported to increase carriage rates, indicating that low socioeconomic standards could be a risk factor for EPE-carriage, but the contrary has also been reported^22–25^. Previous health care admissions, history of antibiotic use prior to admission and ongoing hospitalization are, on the other hand, often reported to be associated with increased fecal carriage rates and are well-known risk factors for EPE-disease^14,15^. Though not conclusive we saw similar trends especially for young children admitted to the zonal referral hospital, which may be factors influencing the higher rates at admissions to this hospital as compared to the other sites studied. A significantly higher carriage rate at admission to the tertiary hospitals is also in agreement with previous studies^2^. In age-adjusted multivariate analysis, only children admitted in the intensive care unit had higher odds of EPE carriage similar to previous findings^4,26,27^. We did not investigate risk factors associated with EPE clinical infections at admission, simply due to the fact that these infections were so rare.

The overall EPE carriage rate was similar to that from other studies of children in a range of countries and settings from Sub-Saharan Africa^8^ and in accordance with the prevalence of 23% which was reported in colonized healthy children in Laos, Asia^28^. However, the prevalence was lower compared to 34% reported for children in Dar es Salaam, Tanzania^2^, and 33% in Ginea-Bissaue^24^. These studies were conducted in 2010–2011 and it is interesting to note that the carriage rates we present are within the same range or even lower five years later despite the increasing ESBL-pandemic and increasing community spread world-wide including in Tanzania^29^. Furthermore, ESBL-*K. pneumoniae* was considerably more prevalent in both these studies. The included children were younger, and other comorbidities were more prevalent, including higher HIV rates which may explain some of the differences observed^2,24^.

The EPE, both *E. coli* and *K. pneumoniae* were highly resistant to commonly used antibiotics, including trimethoprim-sulfamethoxazole, gentamicin and ciprofloxacin. This is to be expected since these resistance genes often are carried on the same easily transferrable plasmids as the ESBL-gene^18^. Some of the most widespread ESBL-carrying *E. coli* clones part of ST131 are also inherently associated with quinolone-resistance^30^. Multidrug resistance to at least three classes of antibiotics was common. This is also a general finding in EPE, but the rates were comparatively high, which appear to be a common finding in Sub-Saharan Africa^2,3^. The current study found that the antimicrobial resistance rate in EPE was very high to the first line antimicrobial agents commonly used for empirical treatment in our setting such as ampicillin, amoxicillin-clavulanic acid and trimethoprim-sulfamethoxazole. This was also true for non-ESBL-producing *E. coli* and for most antibiotics, there were no differences in terms of resistance rates in EPE in the different settings, which indicate a high over-all antibiotic pressure with these drugs. Amoxacillin and trimethoprim-sulfamethoxazole drugs are widely used. They are relatively cheap to purchase and widely available over the counter in oral form^7^. High trimethoprim-sulfamethoxazole resistance might also be due to high use of this antibiotic as prophylaxis against opportunistic infections associated with HIV which makes it a common available drug to be used by HIV negative individuals as well in our settings. Alarmingly, resistance to meropenem was also noted in a few *E. coli* isolates, however most of these isolates were not multidrug resistant or ESBL-producers. It is important to note that this resistance mechanism was not further investigated.

There are a number of limitations to this study. From only 73% (66/90) of colonized children with EPE, a urine samples were obtained in due to confirm or exclude bacteriuria why a potential UTI in these children cannot be excluded. However, no clinical signs indicating UTI was recorded. Only one fecal sample was taken per child, and we cannot exclude that EPE-colonization could have been missed. In addition, we limited EPE to *E. coli* and *K. pneumoniae*as they are the most important and most prevalent EPE pathogens globally. There are isolates of other Gram-negative species that occasionally may carry ESBLs but in general they are rare findings within the respective species^2,22,23,28,31^.

It would however be of interest to study acquisition during hospitalization in this milieu, considering the reduced possibilities of infection control measures and high antibiotic use in general. A recent study from Tanzania has clearly shown high intra-hospital transmission rates in the neonatal unit^32^.

One great benefit of this study was the knowledge exchange and introducing quality controlled antibiotic susceptibility determination including that of ESBL-detection at the routine clinical bacteriology laboratory at KCMC as all analyses were performed in this laboratory. This will hopefully increase the possibility of future clinical studies and better guidance in the use of antibiotics for clinical care in the present settings.

## Conclusion

This is one of few studies from Sub-Saharan Africa assessing the prevalence of ESBL producing *E. coli* and *K. pneumoniae* in young febrile children admitted at three different health care levels within one region. Clinical EPE-infections as confirmed by culture were very rare, despite rather high carriage rates especially in the youngest children and those admitted to the tertiary hospital. No sociodemographic factor could be correlated to EPE-carriage. Patients admitted to ICU and to the tertiary hospital were more likely to carry EPE. Higher rates were also observed in those with a previous history of antibiotic treatment, but this difference was not significant after adjustment for age. Very high resistance to first line antibiotics such as ampicillin, amoxicillin-clavulanic acid and trimethoprim-sulfamethoxazole in the fecal EPE as well as the non ESBL-producing *E coli* isolates is a matter of concern as well as high resistance to gentamicin and ciprofloxacin in EPE. Resistance to meropenem in fecal *E. coli* was also noted though rare in the EPE-isolates.

In the present study we found alarmingly high resistance rates to the most used antibiotics in all levels of the health care system. This demands an urgent need of antibiotic stewardship programs, not only in the hospitals, but also in the primary health care. A continuous high use of antibiotics will soon render these drugs ineffective against infections caused by common bacterial agents, including *E. coli*. For children in advanced care the need of clinical culture sampling in case of suspected infection becomes obvious and needs to be enforced. This study clearly demonstrates that local bacteriological diagnostics, including ESBL-identification and antibiotic susceptibility testing can be implemented in the routine diagnostics in resource-limited settings.

## Data Availability

The datasets used and/or analyzed during the current study are available from the corresponding author on reasonable request.
